# Prenatal Cannabis Exposure Shaping Altered Brain Connectivity: Neural Correlates of Cognitive and Mental Health Variability in Offspring

**DOI:** 10.21203/rs.3.rs-8545326/v1

**Published:** 2026-01-20

**Authors:** Zening Fu, Kent Hutchison, Anika Guha, Jing Sui, Vince Calhoun

**Affiliations:** Georgia Institute of Technology, Emory University and Georgia State University; Tri-Institutional Center for Translational Research in Neuroimaging and Data Science (TReNDS): Georgia State University, Georgia Institute of Technology, and Emory University

## Abstract

Emerging evidence from both human and preclinical research indicates that cannabis use during pregnancy can influence offspring neurodevelopmental outcomes. Δ^9^-Tetrahydrocannabinol (THC), the psychoactive compound in cannabis, permeates the placental barrier and modulates the endocannabinoid system, a critical regulator of neurodevelopmental processes. Although converging findings suggest that prenatal cannabis exposure (PCE) is associated with adverse cognitive and mental health outcomes in offspring, the neurobiological mechanisms underlying these associations—particularly in terms of large-scale functional brain network organization—remain poorly understood. In this large-scale cross-sectional study, we leveraged baseline data from the ongoing longitudinal Adolescent Brain Cognitive Development (ABCD) Study, which enrolled 11,875 children across 22 research sites. We examined the effects of PCE, occurring both before and after maternal awareness of pregnancy, on offspring psychopathology and cognitive performance. Resting-state functional MRI data were analyzed using the NeuroMark framework, enabling the identification of individualized intrinsic connectivity networks (ICNs) and estimation of functional network connectivity (FNC) among them. Associations between prenatal exposure, behavioral outcomes, and functional connectivity were assessed using linear mixed-effects models, controlling for a comprehensive set of familial, pregnancy-related, and child-specific covariates. Among 10,836 children (female/male = 5,194/5,642; mean age = 9.96 ± 0.62 years), 754 (6.96%) were prenatally exposed to cannabis. Compared with non-exposed peers, exposed children exhibited higher levels of psychopathology and poorer cognitive performance, except composite fluid cognition (Cohen’s *d* = − 0.1393 ~ 0.2451, false discovery rate [FDR]–corrected *p* < .05), consistent with prior reports linking PCE to adverse developmental outcomes. Importantly, prenatal exposure was associated with alterations in FNC that significantly overlapped with neurofunctional correlates of both mental health symptoms (positive correlations between *t*-statistics, *r*= 0.0641 ~ 0.5993, FDR-corrected *p* < .05) and cognitive performance (negative correlations, *r* = − 0.5438 ~ − 0.6665, FDR-corrected *p* < .05). These findings provide novel evidence that PCE is associated with altered large-scale brain network connectivity, which in turn relates to both cognitive and mental health outcomes in late childhood. The overlapping neurofunctional correlates of exposure and behavioral outcomes suggest that THC’s interaction with the endocannabinoid system may disrupt the maturation of functional brain networks, providing a potential mechanistic link between prenatal exposure and neurodevelopmental vulnerability.

## Introduction

As societal attitudes and legislative policies regarding cannabis use continue to liberalize ^1^, cannabis has become the most widely consumed illicit substance globally since 2016 ^2^. Its increasing recreational use, along with claims of therapeutic benefits, has led some women to view cannabis as a relatively safe choice during pregnancy. However, public health recommendations clearly advise against cannabis use before conception, during pregnancy, and throughout breastfeeding ^3^. Unfortunately, awareness of the neurodevelopmental risks associated with prenatal cannabis exposure (PCE) remains quite low.

Cannabis has emerged as the most commonly used recreational drug among pregnant individuals ^3,4^. A nationally representative survey in the United States found that the prevalence of past-month cannabis use during pregnancy more than doubled from 3.4% in 2002 to 7.0% in 2017 ^5^. Biological assays indicate even higher actual exposure rates, suggesting significant underreporting in self-reported measures ^6^. Given the increasing prevalence of PCE and accumulating evidence linking prenatal exposure to disruptions in neurodevelopment among offspring, such as changes in cognition and emotional regulation, the U.S. Surgeon General has reiterated that cannabis use during pregnancy and lactation should be strictly avoided ^7^.

The prenatal period constitutes a critical window for brain and behavioral development, during which foundational neurobiological processes shape long-term cognitive, emotional, and psychiatric outcomes in offspring ^8^. Although the developmental consequences of prenatal exposure to substances such as tobacco and alcohol have been extensively characterized, the effects of PCE remain comparatively understudied ^9^. The endocannabinoid (eCB) system, which is active from the early stages of fetal development, plays an essential role in orchestrating neurodevelopmental processes, including neuronal proliferation, migration, differentiation, axonal guidance, and synaptogenesis across circuits subserving cognition, emotion, and reward ^10^. The principal psychoactive constituent of cannabis, Δ^9^-tetrahydrocannabinol (Δ^9^-THC), is a highly lipophilic molecule that readily crosses the placental barrier and binds to cannabinoid receptors (CB_1_ and CB_2_). This exogenous activation can perturb eCB signaling homeostasis, thereby disrupting the tightly regulated processes that govern fetal brain maturation ^11^. Converging evidence from both human and preclinical studies indicates that PCE induces neurochemical and molecular alterations, including dysregulation of dopaminergic D2 receptor signaling, which may increase susceptibility to later-life neuropsychiatric disorders ^12^. Additionally, Δ9-THC exposure has been shown to impair serotonergic neurotransmission, reducing central serotonin levels and contributing to affective dysregulation and elevated risk for mood and anxiety disorders ^10^. PCE is clinically associated with atypical neurodevelopmental trajectories, encompassing deficits in cognitive functioning, behavioral control, and emotional regulation, as well as increased risk for psychosis-spectrum and externalizing symptoms in later childhood and adolescence ^9,13–15^.

Recent advances in neuroimaging have enabled a more precise characterization of the neural substrates underlying PCE, shedding light on how such exposure may alter the developmental trajectory of large-scale brain networks ^16–18^. Functional magnetic resonance imaging (fMRI) and functional connectivity analyses have become indispensable for delineating macroscale network architecture and identifying neurobiological signatures of risk that may forecast cognitive, emotional, and behavioral dysregulation later in life ^19–21^. Findings from resting-state fMRI (rs-fMRI) studies consistently reveal that PCE is associated with disruptions in intrinsic functional connectivity across several core neurocognitive systems, including the striatal ^22^, cerebellar ^23^, salience ^24^, ventral attention ^24^, and cognitive control networks ^25^. These large-scale networks form the backbone of executive control, emotional regulation, and reward processing—domains that are particularly sensitive to perturbations during neurodevelopment ^26,27^. Altered connectivity within and between these systems suggests that PCE may interfere with the maturation of functional integration and hierarchical specialization of neural circuits that support adaptive cognitive and affective processing. However, most existing studies have examined behavioral outcomes and brain alterations in isolation, offering a fragmented view of how prenatal exposure reshapes the brain’s functional topology linked with derailed mental and cognitive health. A more comprehensive, systems neuroscience framework is needed to understand how these distributed connectivity disturbances collectively mediate the link between PCE and neuropsychiatric vulnerability, particularly in relation to psychosis-spectrum symptoms and cognitive dysfunction. Considering that mental health and cognitive abilities are emergent properties of the brain’s functional organization, the convergence of PCE-related neurofunctional alterations and behaviorally relevant network dynamics may delineate a mechanistic pathway through which prenatal cannabis exposure confers risk for atypical neurodevelopment.

To address critical gaps in understanding the neurobiological consequences of PCE, we employed a novel hybrid independent component analysis (ICA) framework, NeuroMark ^28,29^, applied to data from the Adolescent Brain Cognitive Development (ABCD) Study ^30^. This large-scale, population-based cohort includes over 11,000 children with comprehensive behavioral, psychiatric, and neurocognitive assessments ^31^, offering an unprecedented opportunity to characterize whole-brain functional network connectivity (FNC) alterations associated with PCE and to examine their associations with offspring mental health and cognitive outcomes. The innovation of this study is twofold. First, we utilized a data-driven, individualized network-mapping approach across 41,703 fMRI scans from 11,170 participants to derive robust large-scale connectivity patterns. Unlike conventional region-of-interest (ROI) or hypothesis-driven approaches, the NeuroMark framework extracts scan-adaptive functional networks spanning nearly the entire gray matter, guided by the validated NeuroMark Functional 1.0 template. This approach preserves the flexibility and sensitivity of ICA to inter-individual variability while minimizing methodological biases such as ROI misalignment and inter-subject information leakage that can distort network estimation. Consequently, this analytic framework enables precise detection of developmentally specific FNC signatures characteristic of the preadolescent period. Second, extending beyond prior research primarily focused on behavioral associations, we aimed to elucidate the neurofunctional perturbations that mechanistically link PCE to variation in cognitive and mental health outcomes. We hypothesized that PCE would be associated with altered FNC patterns reflecting disrupted large-scale network integration and segregation, particularly involved in neural systems subserving cognitive control, affective regulation, and reward processing. These connectivity alterations were expected to correlate positively with increased psychopathology risk and negatively with cognitive abilities, delineating a potential neurodevelopmental pathway through which prenatal cannabis exposure contributes to enduring alterations in brain function and behavior.

## Methods

### Flowchart of FNC analysis of prenatal cannabis exposure and offspring outcomes

**Figure 1:**
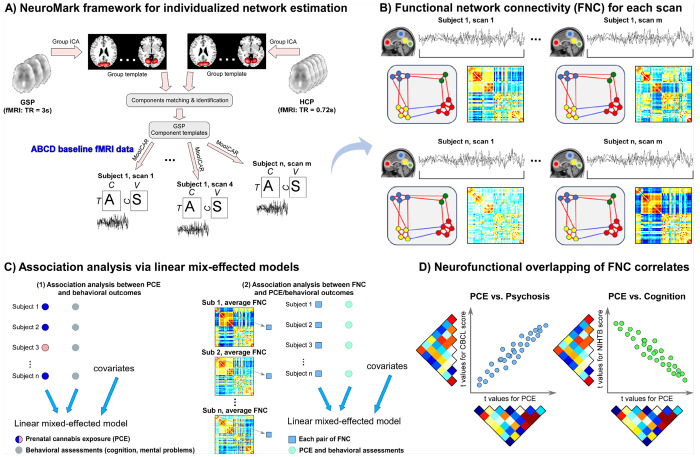
Flowchart for functional network connectivity (FNC) analysis of prenatal cannabis exposure (PCE) and offspring outcomes. **A)** The NeuroMark framework is applied to extract robust and spatially comparable intrinsic connectivity networks (ICNs) and their corresponding time courses (TCs) from the Adolescent Brain Cognitive Development (ABCD) dataset. **B)** FNC matrices are computed from the temporal correlations among ICN TCs for each scan, and subject-level FNC profiles were derived by averaging across within-subject scans. **C)**Linear mixed-effects models (LMMs) are used to assess associations between PCE and offspring behavioral measures, as well as between individual FNC pairs, PCE, and behavioral outcomes. **D)** To identify shared neurofunctional substrates, t-statistic maps from the FNC–PCE and FNC–behavioral models are correlated, quantifying the degree of overlapping brain network alterations linking PCE to offspring cognitive and mental health outcomes.

Figure 1 depicts the analytical flowchart investigating the relationships between FNC, PCE, and offspring mental and cognitive outcomes. The analysis began with the application of the NeuroMark framework, which employs a rigorously validated group-level component template to derive individualized intrinsic connectivity networks (ICNs) that are spatially and functionally comparable across scans and participants. For each scan, FNC matrices were constructed by computing pairwise temporal correlations among ICN time courses (TCs). To generate a stable and reliable subject-level representation, FNC matrices were averaged across all available scans for each individual, producing a comprehensive individualized FNC profile. Subsequently, linear mixed-effects models (LMMs) were implemented to examine (1) associations between PCE and offspring behavioral outcomes, and (2) associations between FNC, PCE, and these behavioral measures. To identify shared neurofunctional substrates underlying these associations, we computed correlations between the *t*-statistic maps derived from the FNC–PCE models and those from the FNC-behavioral models, encompassing both mental health and cognitive domains. This integrative approach enabled quantification of the overlapping large-scale brain network architecture through which PCE may exert its influence on neurodevelopment, potentially linking prenatal exposure to alterations in cognitive and emotional functioning during late childhood.

### Dataset and participants

We utilized neuroimaging and behavioral data from the ABCD Study, release 6.0, to examine the effects of PCE on functional brain connectivity, cognitive performance, and mental health outcomes in late childhood. The ABCD Study offers a comprehensive framework for developmental neuroscience, incorporating a wide range of standardized assessments, including neurocognitive testing, MRI imaging, physical health indices, and detailed psychosocial and environmental measures. This extensive collection of phenotypic and neurobiological data enables a multidimensional investigation of how early exposures influence neurodevelopmental trajectories across cognitive, emotional, and behavioral domains ^32^. All procedures were conducted in accordance with ethical standards approved by the Institutional Review Board (IRB). Centralized IRB oversight was provided by the University of California, San Diego, with additional local IRB approvals obtained at each participating site. Parents or legal guardians provided written informed consent, and all participating children provided assent. The analyses focused on baseline data (mean age = 9.96 ± 0.63 years), including participants with both complete neuroimaging and behavioral assessments.

Resting-state fMRI data and associated tabulated information were obtained from the National Institute of Mental Health (NIMH) Brain Development Cohorts (NBDC) Data Hub (https://www.nbdc-datahub.org/). This hub provides secure, centralized access to longitudinal neuroimaging, behavioral, and genomic data from the ABCD Study. The fMRI data were preprocessed using the FMRIB Software Library (FSL v6.0) ^33^ and Statistical Parametric Mapping (SPM12) ^34^, implemented in MATLAB R2024b. Preprocessing procedures consisted of the following steps: 1) removal of the initial 10 volumes to allow for magnetic field stabilization, 2) rigid-body motion correction, 3) distortion correction, 4) spatial normalization to the Montreal Neurological Institute (MNI) standard space, and 5) spatial smoothing with a 6 mm full-width at half-maximum (FWHM) Gaussian kernel. Following preprocessing, we applied quality control (QC) steps to verify the integrity of the imaging data. We assessed each scan based on the spatial correlation between individual and group brain masks. Scans that fell below predefined correlation thresholds were excluded from further analysis. Detailed descriptions of the preprocessing pipeline and QC criteria are provided in the Supplementary Materials, under the sections “Data Preprocessing” and “Imaging Quality Control.”

### Assessment of Prenatal Exposure

Prenatal exposure to substances was determined based on maternal self-report in questionnaire surveys, which documented exposure to medications, drugs, alcohol, and tobacco ^35^. From the developmental history table (ph_p_dhx.tsv), we focused on two specific questions: 1) “Before the biological mother knew she was pregnant, but while she might have been pregnant with this child, did she use marijuana?” and 2) “After the biological mother became aware of her pregnancy, did she use marijuana?” Children were considered PCE if their biological mothers reported marijuana use at either time point. Additionally, information regarding prenatal exposure to alcohol and tobacco was extracted and included as covariates in subsequent analyses to control for potential confounding effects. Note that, at the four-year follow-up session, baseline responses were pre-populated in the questionnaire, and research staff verified and updated this information as necessary. Consequently, the exposure variables were adjusted based on the four-year follow-up data.

### Assessments of Children’s Mental Health and Cognitive Functioning

Children’s mental health was assessed using the Child Behavior Checklist (CBCL, mh_p_cbcl.tsv), a standardized parent-report instrument designed to evaluate a broad spectrum of emotional and behavioral problems, including internalizing and externalizing symptoms, as well as syndrome-specific and DSM-oriented scales ^36,37^. In this study, we analyzed t-scores across 20 CBCL scales encompassing behavioral and emotional functioning. Specifically, the six DSM-oriented scales included: 1) Attention-Deficit/Hyperactivity Disorder (ADHD), 2) Anxiety Problems, 3) Conduct Problems, 4) Depressive Problems, 5) Oppositional Defiant Problems, and 6) Somatic Problems. Eleven item-level CBCL scales were also examined: 1) Obsessive-Compulsive Problems, 2) Sluggish Cognitive Tempo, 3) Stress Problems, 4) Aggressive Behavior, 5) Anxious/Depressed, 6) Attention Problems, 7) Rule-Breaking Behavior, 8) Social Problems, 9) Somatic Complaints, 10) Thought Problems, and 11) Withdrawn/Depressed. Additionally, three composite scales were considered: Internalizing Problems, Externalizing Problems, and Total Problems. The CBCL has been extensively utilized in neuroscience research to investigate associations between children’s behavioral and emotional profiles and brain organization, providing a validated framework for linking symptomatology to large-scale neural brain networks ^38–40^.

Children’s cognitive functioning was assessed using uncorrected scores from the National Institutes of Health Toolbox Cognition Battery (NIHTB; nc_y_nihtb.tsv), a standardized, computerized neurocognitive assessment designed to evaluate core domains of cognitive development ^41^. The NIHTB includes seven component tasks: 1) Picture Vocabulary, 2) Flanker Inhibitory Control and Attention, 3) Picture Sequence Memory, 4) Dimensional Change Card Sort, 5) Pattern Comparison Processing Speed, 6) Oral Reading Recognition, and 7) List Sorting Working Memory. In addition to individual task scores, three composite indices—Crystallized Cognition, Fluid Cognition, and Total Cognition—were derived to capture higher-order dimensions of cognitive performance. The NIHTB has been extensively utilized in large-scale developmental and neuroimaging cohorts, such as the ABCD Study, to investigate the neural substrates of cognitive variability, providing a validated framework for linking behavioral measures of cognition to imaging neuroscience ^42,43^.

### NeuroMark Framework and Functional Network Connectivity

The rsfMRI data were analyzed using the NeuroMark framework, implemented via the Group ICA of fMRI Toolbox (http://trendscenter.org/software/gift) ^44^ with the NeuroMark v1.0 functional template (http://trendscenter.org/data) ^28^. NeuroMark addresses key limitations of conventional atlas-based and decomposition-based approaches by integrating a spatially constrained ICA with a robust, population-derived functional template. This hybrid approach enables the extraction of individualized ICNs that are spatially comparable across participants and scans, while preserving the advantages of data-driven network discovery sensitive to individual variability. Our research has demonstrated the reproducibility and generalizability of NeuroMark-derived templates across the lifespan ^29^, from infancy to older adulthood, supporting its applicability to the ABCD study. Using this framework, we extracted scan-specific ICNs and their corresponding time courses (TCs), aligned to 53 functional networks spanning seven canonical domains: subcortical (SC), auditory (AUD), sensorimotor (SM), visual (VS), cognitive control (CC), default mode (DM), and cerebellar (CB).

TCs derived from each scan were subjected to standardized post-processing procedures to minimize noise and non-neuronal confounds. These steps included: 1) detrending to remove linear, quadratic, and cubic trends; 2) regression of six rigid-body motion parameters and their first temporal derivatives; 3) detection and exclusion of artifact-contaminated time points; and 4) temporal band-pass filtering (0.01–0.15 Hz) to isolate low-frequency fluctuations characteristic of resting-state connectivity. FNC was computed as the Pearson correlation between all pairs of denoised TCs, yielding a 53 × 53 symmetric connectivity matrix for each scan. For participants with multiple baseline scans, individual FNC matrices were averaged to derive a stable subject-level mean connectivity profile. The off-diagonal elements of these matrices, corresponding to 1,378 unique pairwise connections, served as the primary neuroimaging features for subsequent statistical analyses.

### Associations between Connectivity, Prenatal Exposure, and Offspring’s Outcomes

Statistical analyses were performed using linear mixed-effects models (LMMs) to account for the hierarchical structure of the data, with family membership nested within site ^19,20^. This modeling approach mitigates the influence of non-independence arising from shared familial and site-level factors. LMMs were first applied to examine associations between PCE and children’s behavioral and cognitive outcomes, as measured by the CBCL and NIHTB, respectively. Each CBCL or NIHTB score served as the dependent variable, with PCE (0 = no exposure; 1 = exposure) specified as a fixed-effect predictor. Covariates included child age, sex, and prenatal exposure to alcohol and nicotine, while family structure and site were modeled as random effects to control for clustering and potential confounds. Children reporting cannabis use were excluded from analyses to eliminate direct exposure effects. To investigate neurobiological functional correlates of PCE and behavioral outcomes, LMMs were conducted using each FNC pair as a fixed-effect predictor, with dependent variables as prenatal exposure status, mental health, or cognitive measures. Model covariance structures mirrored those described above. For each model, the correlation coefficient (r), associated t-statistic, and Cohen’s *d* were estimated to quantify effect magnitude and directionality. Multiple comparisons across behavioral assessments and FNC pairs were corrected using the false discovery rate (FDR) procedure to control for Type I error inflation.

To assess the degree of overlap between the FNC correlates of PCE and those associated with children’s mental health and cognitive functioning, we quantified the correspondence between model-derived effects. Specifically, Pearson’s correlations were computed between the t-statistic map from the PCE–FNC model and those from each of the 20 mental health–FNC models and 10 cognitive function–FNC models, respectively, across all connectivity pairs ^45^.

## Results

### Associations between Prenatal Cannabis Exposure and Mental/Cognitive Problems

QC procedures yielded a total of 10,836 participants with at least one high-quality resting-state fMRI scan from the baseline session, comprising 754 children with PCE and 10,082 non-exposed controls. Participants with inconsistent or ambiguous exposure reports (e.g., combinations of “no exposure” and “not sure”) were excluded to ensure data integrity. Power analysis demonstrated that, assuming moderate measurement variability (~ 5% of the mean signal) and small-to-moderate effect sizes (*r* = 0.10) in brain-wide association studies ^46^, this sample size (*n* = 10,836) provides sufficient statistical power (1–*β* = 0.80) to detect robust correlations at a Type I error threshold of *α* = 0.05.

**Figure 2:**
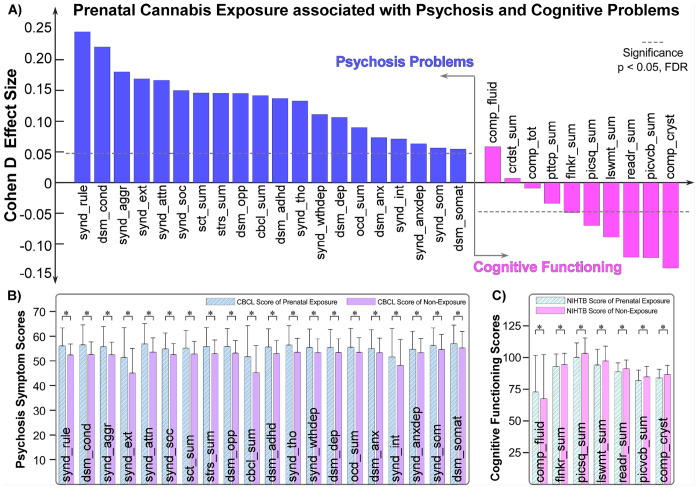
Associations between prenatal cannabis exposure (PCE) and offspring’s mental health and cognitive functioning during late childhood. **A)** PCE is associated with elevated psychopathology scores on the Child Behavior Checklist (CBCL) and generally reduced cognitive performance on the NIH Toolbox Cognition Battery (NIHTB) among children aged 9–10 years. Effect sizes (Cohen’s d) estimated from linear mixed-effects models are shown for each assessment. **B)** Group comparisons of CBCL psychopathology scales between children with and without PCE reveal consistently higher symptom severity among exposed children, with the most pronounced difference observed for the Rule-Breaking Behavior scale. Significant group differences are identified across all 20 CBCL scales. **C)** Comparisons of NIHTB cognitive performance indicate generally lower cognitive functioning among exposed children, with the largest difference observed for the composite crystallized cognition score. A reverse effect is noted for the composite fluid cognition score, where exposed children demonstrate modestly better performance. Significant group differences are observed in 7 NIHTB measures. Bars represent mean scores, and error bars indicate standard deviations. * indicates significance with *p*< 0.05, False discovery rate (FDR)-corrected.

Analysis of the behavioral assessments revealed significant associations between PCE and children’s mental health problems and cognitive functioning. Children with PCE exhibited significantly higher scores across all 20 CBCL scales compared to non-exposed peers (Cohen’s *d* = 0.0546 to 0.2451, *p* = 4.52 × 10^−3^ to 5.71 × 10^−37^, FDR-corrected significance; Fig. 2A). The largest effect was observed for the Rule-Breaking Behavior scale (Cohen’s *d* = 0.2451, *p* = 5.71 × 10^−37^; Fig. 2B), where exposed children (56.15 ± 7.26) demonstrated higher symptom scores than non-exposed children (52.45 ± 4.48). The smallest effect was observed for the DSM-oriented Somatic Problems scale (Cohen’s *d* = 0.0546, *p* = 0.0045; Fig. 2B), with exposed children (57.06 ± 7.52) exhibiting slightly higher scores than non-exposed children (55.35 ± 6.55). These findings indicate that PCE is associated with elevated behavioral and emotional problems in offspring during late childhood, with the greatest impact observed in domains related to rule-breaking and externalizing behaviors.

In contrast, among the 10 cognitive assessments, children with PCE exhibited significantly lower performance in 6 measures relative to non-exposed peers (Cohen’s *d* = − 0.0490 to − 0.1393, *p* = 0.0112 to 6.17 × 10^−13^, FDR-corrected significance; Fig. 2A). The most pronounced effect was observed for the Composite Crystallized Cognition score (Cohen’s *d* = − 0.1393, *p* = 6.17 × 10^−13^; Fig. 2C), indicating that exposed children (83.89 ± 6.71) performed significantly worse than non-exposed children (86.66 ± 7.01) on tasks requiring acquired knowledge and verbal comprehension. Interestingly, an opposite effect emerged for the Composite Fluid Cognition score (Cohen’s *d* = 0.0585, *p* = 0.0023; Fig. 2C), where exposed children (72.83 ± 28.80) showed higher performance compared to non-exposed peers (67.41 ± 34.66) on tasks reflecting novel problem-solving and reasoning independent of prior learning. Collectively, our findings suggest that PCE is predominantly associated with reduced cognitive performance across most domains, except for fluid cognition, which showed modest enhancement.

### Correlate FNC Anatomy with Prenatal Cannabis Exposure

The NeuroMark framework parcellated the brain into 53 ICNs, organized into seven canonical functional domains based on their anatomical and putative functional roles (Table S1; Fig. S1). Specifically, the parcellation included five ICNs within the SC domain, two within the AUD domain, nine within the SM domain, nine within the VS domain, 17 within the CC domain, seven within the DM domain, and four within the CB domains.

**Figure 3:**
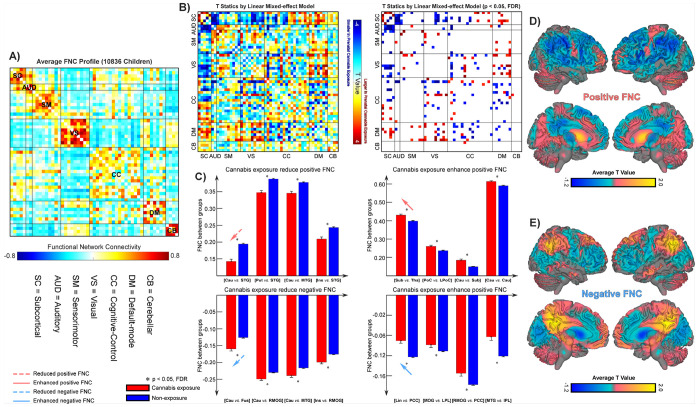
Prenatal cannabis exposure (PCE) and functional brain alterations during late childhood. **A)** Functional network connectivity (FNC) was computed using Pearson correlation coefficients between network time courses. The group-averaged FNC matrix demonstrates clear modular organization within and between canonical functional domains, confirming the robustness of the NeuroMark framework. **B)** Associations between PCE and FNC are assessed using linear mixed-effects models. The corresponding *t*-values are displayed, with significant connections surviving false discovery rate (FDR) correction (*p* < 0.05) highlighted. **C)** FNC pairs exhibiting the most pronounced increases and decreases in positive and negative connectivity are presented, illustrating the directionality and magnitude of network-level alterations associated with PCE. **D)** Mean *t*-values were computed across positive and negative FNC pairs for each network, revealing global patterns of increased and decreased connectivity across functional systems in children prenatally exposed to cannabis.

The FNC analysis examining the relationship between PCE and offspring’s FNC demonstrated that children’s functional network organization during late childhood exhibits widespread and diverse alterations linked to cannabis use during pregnancy (Fig. 3B). Notably, children with PCE exhibited increased FNC within the SC and SM domains, as well as between the DM and SC/sensory domains. In contrast, decreased FNC was observed within the DM domain, between SC and sensory domains, and between DM and CC domains. The FNC between SC and CC, between VS and CC, and within the CC domain demonstrated heterogeneous alterations, with both increases and decreases detected among PCE children. Figure 3C highlights the most pronounced increases and decreases in FNC for positive and negative connections. For instance, the positive FNC between the caudate (Cau) and superior temporal gyrus (STG) exhibited the largest decrease. In contrast, the positive FNC between the subthalamus (Sub) and thalamus (Tha) showed the largest increase in exposed children. Meanwhile, the negative FNC between the Cau and fusiform gyrus (Fus) demonstrated the largest reduction, while the negative FNC between the lingual gyrus (Lin) and posterior cingulate cortex (PCC) showed the largest increase. To further visualize the network-level alterations, we computed the mean t-values across positive and negative FNC pairs for each network, as shown in Fig. 3D and 3E. The results revealed that subcortical networks exhibited enhanced positive connectivity and attenuated negative connectivity in children with PCE, whereas the precentral/postcentral central gyrus and posterior default-mode networks displayed the opposite trend.

### Overlap of Connectivity Alterations Linking Prenatal Cannabis Exposure to Mental and Cognitive Health

The FNC analysis on the CBCL-derived behavioral symptom scores revealed overlapping FNC alterations with those identified in relation to PCE. Specifically, increased FNC within SC and SM domains, as well as between the DM and sensory domains, is associated with higher CBCL psychosis scores. In contrast, decreased FNC between the SC and sensory domains—particularly involving the VS domain—was linked to more psychosis symptom scores. Connectivity involving the CC domain exhibited heterogeneous alterations, with both increases and decreases associated with CBCL mental health scores.

**Figure 4:**
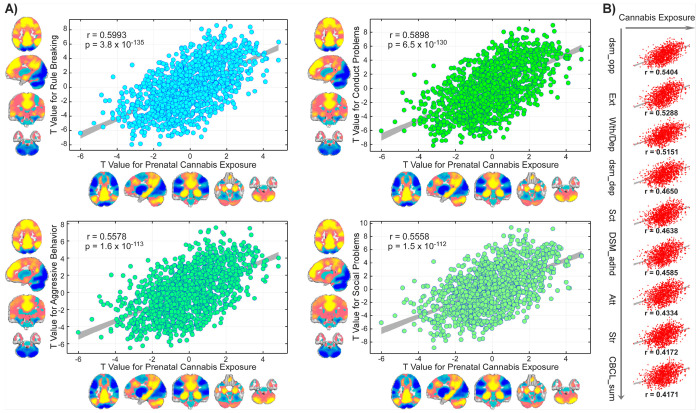
Overlap between prenatal cannabis exposure (PCE)–related functional connectivity alterations and offspring mental health during late childhood. The positive correlations between PCE- and Child Behavior Checklist (CBCL)-related functional network connectivity (FNC) patterns align with their behavioral relationship. **A)** Four CBCL syndrome scales for—rule-breaking behavior, conduct problems, aggressive behavior, and social problems—show the strongest positive correspondence in t-statistics with PCE-related FNC alterations (*r* = 0.5558 to 0.5993, *p* < 1 × 10^−10^). Network-level mapping illustrates the spatial convergence of these associations across large-scale brain systems, except for the cerebellum. **B)** Nine additional CBCL syndrome scales exhibit positive correlations with PCE-related *t*-statistics exceeding *r* = 0.40, indicating broad convergence of PCE-linked neural alterations with individual differences in mental problems.

To quantitatively assess the overlap between PCE–related and CBCL-related FNC patterns, we computed correlations between the corresponding *t*-statistics derived from each association analysis. This analysis revealed generally positive correlations (*r* = 0.0641 to 0.5993, *p* = 0.0255 to 3.84 × 10^−135^, FDR-corrected significance; Fig. 4A–B), consistent with the observed spatial overlap in the brain. The positive correlations of connectome *t* weights between PCE and CBCL scores aligned with their behavioral relationship. Among all behavioral domains, the CBCL rule-breaking behavior scale exhibited the strongest correspondence with PCE–related FNC alterations (*r*= 0.5993, *p*= 3.84 × 10^−135^). The next strongest correlations were observed for conduct problems (*r* = 0.5898, *p* = 6.50 × 10^−130^), aggressive behavior (*r* = 0.5578, *p* = 1.59 × 10^−113^), and social problems (*r* = 0.5558, *p* = 1.53 × 10^−112^). Network-level visualization further supported these findings, revealing convergent FNC alterations across domains implicated in both PCE and mental health dysregulation, except the cerebellar networks. As shown in Fig. 4B, correlations exceeding *r*= 0.4—such as DSM-Oriented Oppositional Defiant Problems and CBCL Externalizing Problems—highlight consistent connectivity perturbations linking PCE with children’s mental health vulnerabilities.

The FNC analyses examining associations with cognitive performance revealed spatially overlapping but directionally divergent patterns relative to those observed for PCE. Analyses focused on the seven NIHTB measures that showed significant associations with PCE, of which six were lower and one was higher among exposed children. For the cognitive domains negatively impacted by PCE, greater cognitive performance was associated with decreased FNC within the SC and SM domains, as well as between the DM and sensory networks. In contrast, increased FNC between the SC and sensory domains—particularly involving VS networks—was linked to enhanced cognitive function. FNC of the CC domain exhibited mixed associations, with both increases and decreases related to performance variability. Moreover, strengthened connectivity between the SM and CB domains was positively associated with cognitive abilities, in the opposite direction of their relationship with PCE. This finding suggests opposing neural mechanisms underlying cognitive resilience versus the effects of PCE.

**Figure 5:**
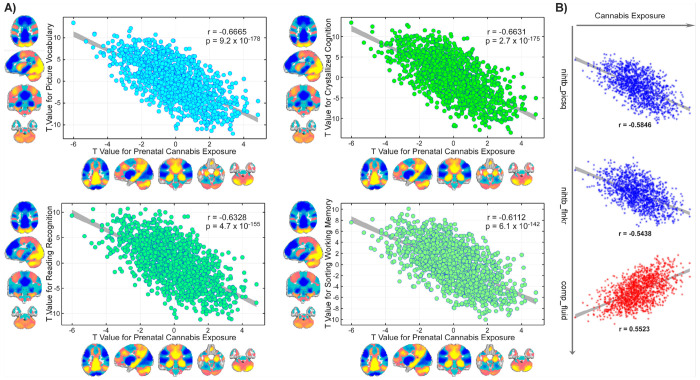
Overlap between prenatal cannabis exposure (PCE)–related functional connectivity alterations and offspring cognitive functioning during late childhood. The correlations between PCE- and National Institutes of Health Toolbox Cognition Battery (NIHTB)-related functional network connectivity (FNC) patterns align with their behavioral relationship. **A)** Four NIHTB scores for—picture vocabulary, composite crystallized cognition, oral reading recognition, and list sorting working memory—show the strongest negative correspondence in *t*-statistics with PCE-related FNC alterations (*r* = −0.6112 to −0.6665, *p* < 1 × 10^−10^). Network-level mapping illustrates the spatial convergence of these associations across large-scale brain systems, except for the cerebellum. **B)** Two additional cognitive measures exhibit negative correlations, whereas one (composite fluid cognition) demonstrates a positive correlation in *t*-statistics with PCE–related FNC alterations, underscoring the convergent yet directionally distinct connectivity signatures linking PCE to cognitive performance in late childhood.

The correlations between PCE- and NIHTB-related FNC patterns align with their behavioral relationship. For those cognitive abilities negatively impacted by the PCE, strong negative correlations were observed (*r* = − 0.5438 to − 0.6665, *p* = 6.72 × 10^−178^ to 9.23 × 10^−178^; Fig. 5A–B), consistent with the reverse overlap in the brain. The most pronounced effects were observed for picture vocabulary (*r* = − 0.6665, *p* = 9.23 × 10^−178^), composite crystallized cognition (*r* = − 0.6631, *p* = 2.71 × 10^−175^), oral reading recognition (*r* = − 0.6328, *p* = 4.72 × 10^−155^), and list sorting working memory (*r* = − 0.6112, *p* = 6.14 × 10^−142^). Two additional cognitive measures, picture sequence memory and flanker inhibitory control and attention, also showed negative correlations between their FNC *t*-statistics and those derived from PCE. Interestingly, for the cognitive measure, which was higher in children with PCE, the composite fluid cognition exhibited a positive correlation with PCE-related FNC t-statistics (*r*= 0.5523, *p*= 7.45 × 10^−111^; Fig. 5B). Collectively, these results indicate that PCE might be associated with widespread functional connectivity perturbations linked to cognitive development in late childhood.

## Discussion

In this study, we investigated the extent to which prenatal cannabis exposure (PCE) and offspring outcomes converge at the level of large-scale brain networks, leveraging the NeuroMark FNC framework and univariate correlate modeling. Our first finding demonstrates that individual differences in PCE, mental health symptoms, and cognitive functioning are significantly associated with distinct patterns of intrinsic brain connectivity, independent of potential confounding factors. The second key finding reveals substantial overlap in the neural representations underlying PCE, mental health, and cognitive outcomes. Specifically, convergent brain alterations across subcortical, sensory, cognitive control, and default mode systems suggest a disruption of coordinated subcortical–cortical integration due to prenatal exposure, critical for adaptive cognition and emotion regulation. Collectively, these results provide novel evidence that PCE links with widespread reorganization of functional brain networks, which may underlie observed variations in cognitive and behavioral development during late childhood.

### NeuroMark and Robust FNC in Children

Brain functional connectivity, and its network-level representation—functional network connectivity—provides critical insights into intrinsic brain organization and large-scale neural communication ^47,48^. The precise characterization of reliable connectivity features holds substantial promise for elucidating the neurobiological mechanisms underlying psychiatric and neurological disorders and for advancing our understanding of complex human behavior ^49^. Among the existing analytic strategies for brain segmentation, atlas-based and decomposition-based approaches (e.g., ICA) are most commonly employed, yet both exhibit inherent limitations. Atlas-based analyses impose fixed anatomical parcellations across scans and individuals, potentially oversimplifying spatial variability in functional organization ^50,51^. Conversely, decomposition-based methods such as ICA often produce non-correspondent components across datasets or runs, complicating result validation and impeding large-scale biomarker development ^52^. To address these challenges, we developed the NeuroMark framework ^28^, which integrates predefined network templates with data-driven decomposition to derive reproducible and biologically meaningful imaging features. Unlike conventional ICA-based methods, NeuroMark leverages standardized templates to ensure cross-subject correspondence while constraining the solution space to enhance the detection of robust biomarkers. Concurrently, its data-driven feature extraction retains subject-specific variability, thereby improving sensitivity to inter-individual neurobiological differences ^53^.

This study represents the first large-scale investigation (N > 10,000) to employ the NeuroMark framework with the Functional 1.0 template to characterize alterations in children’s FNC associated with PCE. The Functional 1.0 template was originally derived from two extensive healthy adult cohorts, the Human Connectome Project (HCP; mean age = 28.79 years) and the Genomics Superstruct Project (GSP; mean age = 21.54 years), and therefore primarily reflects the canonical organization of young adult brain networks. Despite this adult-based reference, the group-averaged FNC matrix from the present children sample (Fig. 3A) exhibited a clear modular organization consistent with established large-scale functional systems, supporting the robustness and generalizability of the NeuroMark ^54,55^. Interestingly, several connectivity features in the child cohort deviated from those previously reported in the adult literature ^28,54,56,57^. Specifically, children demonstrated attenuated positive coupling among sensory systems—such as between the auditory and sensorimotor, and between the sensorimotor and visual networks—as well as diminished negative connectivity between the sensorimotor and cerebellar domains. Conversely, stronger positive associations were observed between visual and cerebellar regions. These distinctions underscore the sensitivity of NeuroMark to age-specific functional architectures. The framework not only ensures reliable cross-subject correspondence in FNC estimation but also captures the adaptive properties of large-scale brain networks characteristic of the developing brain.

### Prenatal Cannabis Exposure, Perturbation in FNC Associated with Offspring Outcomes

Cannabis has been utilized by humans for millennia, with historical records documenting its diverse applications across medicinal, spiritual, and recreational contexts ^58^. By the 19th century, cannabis had been incorporated into Western pharmacopoeia as an analgesic and sedative; however, regulatory restrictions in the 20th century curtailed its medical and non-medical use due to increasing public health concerns ^59^. In recent decades, the resurgence of scientific inquiry and sociocultural shifts surrounding cannabis legalization have led to a reevaluation of its therapeutic potential and perceived safety. This changing landscape has coincided with a substantial increase in cannabis use across different populations, including among pregnant women ^60^. Women report perceiving cannabis as a relatively safe remedy for managing pregnancy-related symptoms such as stress, anxiety, nausea, and pain, as well as for recreational purposes.

However, accumulating evidence indicates that Δ9-THC, the principal psychoactive component of cannabis, can exert direct effects on the developing fetal brain ^61,62^. Prenatal exposure to Δ^9^-THC has been associated with alterations in neurodevelopmental processes and an increased risk of adverse cognitive and behavioral outcomes in offspring, suggesting enduring neurobiological consequences of in utero cannabinoid exposure. Existing studies have established that PCE might be linked to potential psychiatric hazards and brain alterations in offspring, but the results are inconsistent and disparate. For instance, a review by Bassalov et al. reported that PCE is not associated with an increased risk for autism spectrum disorder (ASD) or the development of psychotic, anxiety, or depressive symptoms ^63^, a finding that contrasts with evidence from large-scale studies linking PCE to a broad spectrum of offspring psychopathology ^9,18^. Other research has indicated that PCE may alter resting-state functional connectivity in specific brain regions, potentially mediating associations with psychosis ^24,64^. However, these findings are heterogeneous and do not elucidate the neurobiological substrates of PCE-related outcomes from the perspective of the brain topological perturbations. A primary finding of the current study is that mental health and cognitive functioning rely on overlapping functional systems in the brain that could possibly be affected by PCE. Specifically, *t*-maps of connectivity patterns extracted from the PCE-FNC model demonstrated highly positive correlations with those from the psychosis-FNC models and negative correlations with those from the cognition-FNC models, aligned with their behavioral relationships between PCE and mental and cognitive outcomes. These findings suggest that connectivity patterns enhanced in children with PCE are associated with an increased risk of psychotic symptoms, whereas stronger connections observed in non-exposed individuals are linked to better cognitive functioning ^65^. Moreover, our analyses across multiple subdomains confirmed that the LMM models identified brain connectivity patterns sensitive to shared neural components underlying both PCE and adverse outcomes among offspring ^66^.

The alterations in FNC associated with PCE within subcortical–sensory–frontal circuits are profoundly modulated by the endocannabinoid system, primarily through the widespread distribution of Cannabinoid Receptor 1 (CB_1_) across these regions ^67,68^. CB_1_ receptors are highly expressed in subcortical structures and thalamic relay nuclei, which serve as critical nodes connecting sensory inputs to frontal cortical areas ^67^. Notably, elevated receptor densities are observed in sensory cortices, particularly the visual and auditory cortices, as well as in frontal regions such as the orbitofrontal cortex (OFC), anterior cingulate cortex (ACC), and prefrontal cortex (PFC) ^69,70^. Functionally, the endocannabinoid system exerts regulatory control over synaptic transmission and plasticity within these circuits, thus maintaining the excitatory-inhibitory balance essential for optimal neural function. This modulation underpins critical processes including sensorimotor integration, reward processing, motivational drive, executive functioning, and emotional regulation—all of which are pivotal to mental health and cognition ^71,72^. Perturbations to cannabinoid signaling—such as those resulting from prenatal Δ^9^-THC exposure—may induce the increased within-domain connectivity coupled with decreased between-domain connectivity. Such perturbations potentially impair communication across subcortical, sensory, and frontal networks, thereby contributing to deficits in cognition and heightened vulnerability to psychiatric problems in affected offspring ^17,23^.

Functional segregation between subcortical and sensory networks encapsulates the degree to which these systems maintain distinct patterns of activity and specialized computational roles, reflecting their contribution to hierarchical neural processing. Subcortical regions are primarily involved in modulating arousal, detecting salient stimuli, emotional regulation, and gating sensory inputs, serving as pivotal nodes for integrating internal states and external stimuli. Conversely, sensory cortices are specialized for modality-specific perceptual encoding, facilitating the accurate representation of sensory information. Appropriate segregation of these networks supports efficient hierarchical processing, whereby behavioral relevance is conveyed through selective filtering, integration, and routing of sensory information to higher-order frontal regions engaged in executive and cognitive functions. Disruptions in this segregation—manifested as reduced connectivity—have been implicated in various neuropsychiatric conditions, indicating impaired communication between sensory-processing regions and subcortical hubs that mediate salience, affective regulation, and gating mechanisms. For instance, in social anxiety disorder, widespread abnormalities in functional network connectivity involve both subcortical and perceptual systems, including sensorimotor, auditory, and visual networks, suggesting disrupted coordination between subcortical salience hubs and sensory cortices ^73^. Similarly, in ASD, diminished thalamo-sensory connectivity correlates with atypical sensory perception and deficits in sensory integration, which may underlie social perceptual impairments characteristic of the condition ^74^. Conversely, in neurotypical populations, increased or more organized thalamo-sensory connectivity has been associated with enhanced cognitive functions such as processing speed, attentional control, and memory consolidation. Resting-state functional connectivity between the thalamus and sensory/motor networks supports sensorimotor efficiency ^75^, enabling subcortical-sensory pathways to facilitate bottom-up sensory inputs that underpin working memory, attentional modulation, and executive processing ^76^. Collectively, these findings suggest that PCE may exert lasting effects on offspring’s brain functional organization, potentially via disruptions in neurodevelopmental pathways, immune signaling, and metabolic processes. Δ^9^-THC, acting as a partial agonist at cannabinoid CB_1_ receptors, could interfere with endogenous endocannabinoid signaling, impair dendritic arborization, modulate synaptic plasticity, and hinder the maturation of large-scale neural networks comprising subcortical, sensory, and frontal components. Such perturbations may increase vulnerability to long-term cognitive deficits, behavioral abnormalities, and psychiatric disorders, emphasizing the critical impact of cannabinoid exposure during sensitive developmental windows.

A prominent disparity between PCE-related FNC and offspring-outcome-related FNC pertains to cerebellar connectivity, which exhibits robust associations with mental health and cognitive development but shows no significant relationship with PCE. Traditionally conceptualized as a motor coordination center dedicated to sensorimotor control, the cerebellum has been increasingly recognized for its broader role in higher-order brain functions ^77^. Evidence from neuroimaging, lesion analyses, and developmental neuroscience underscores the cerebellum’s critical contribution to cognitive, affective, and executive processes, facilitated through extensive reciprocal connections with prefrontal, parietal, limbic, and associative cortical regions ^78,79^. This paradigm shift reflects an evolving understanding of the cerebellum as integral to distributed neural networks that support complex cognition, beyond its traditional motor functions ^80^. Cerebellar dysfunction has also been implicated in a range of psychiatric and neurodevelopmental disorders, given its participation in closed-loop systems that underpin reward processing, error prediction, emotional regulation, and autonomic arousal. Disruption of these circuits can precipitate psychosis-like symptoms even in the absence of overt motor deficits ^81,82^.

Developmentally, the cerebellum differs markedly from subcortical and primary sensory structures; while the latter undergo substantial neurogenesis, laminar differentiation, and thalamocortical innervation predominantly during mid-to-late gestation, the cerebellum’s significant growth occurs postnatally ^83^. Specifically, during infancy, the cerebellum experiences pronounced proliferation of granule cells, synaptogenesis, dendritic arborization, and myelination ^84^, leading to volume increases of two- to three-fold within the first two years of life ^85^. In contrast, subcortical structures and primary sensory cortices undergo substantial neurogenesis, laminar differentiation, and thalamocortical innervation during mid-to-late gestation, resulting in core functional architectures that are largely established before birth ^86^. Given this timeline, it is plausible that the cerebellum, whose development peaks postnatally, may be relatively resilient to the direct teratogenic effects of in utero Δ^9^-THC exposure, which readily crosses the placental barrier and disrupts endogenous endocannabinoid signaling during critical prenatal neurodevelopmental windows. Accordingly, the differential vulnerability—where early-maturing subcortical and sensory systems are more affected than the cerebellum—may reflect the primary neural substrates mediating the long-lasting cognitive, behavioral, and mental health impairments observed following PCE. Future research should aim to delineate the specific roles of PCE-sensitive brain regions in the emergence of particular cognitive deficits and psychiatric vulnerabilities, thereby enhancing our understanding of the neurobiological mechanisms through which PCE perturbs neurodevelopmental trajectories.

### Limitations and Future Directions

Several limitations warrant consideration. Although the large ABCD sample strengthens the reliability of our findings, the observed effect sizes were modest (maximum |Cohen’s d| = 0.26), limiting immediate clinical relevance. Nonetheless, this aligns with emerging evidence that true brain–behavior associations are typically small and require large, high-quality datasets for robust detection ^87,88^. Similar to advances in genomics, progress in neuroimaging will depend on large-scale, standardized data and rigorous analytic frameworks. Future work integrating stratified designs and multivariate or multimodal analyses may improve the interpretability and translational value of these subtle but meaningful effects ^46,89^.

Second, the present study concentrated on functional connectivity alterations associated with PCE and offspring mental and cognitive outcomes. Yet, accumulating evidence indicates that PCE also affects brain structure, including gray and white matter development ^90,91^. Prior studies have typically examined structural and functional alterations in isolation, limiting insight into their joint contributions to neurodevelopmental risk. A more comprehensive understanding of PCE’s neural impact will require multimodal imaging approaches that integrate structural and functional data within unified analytic frameworks. Leveraging advanced data fusion and mediation modeling techniques ^92^ could clarify how cannabis-related neural alterations across modalities collectively mediate cognitive and behavioral outcomes in exposed children.

Third, this study utilized only baseline data from the ABCD cohort, which constrains causal inference and limits insight into the developmental trajectory of PCE-related effects. The cross-sectional design precludes the determination of whether observed neural and behavioral alterations reflect transient adaptations or stable neurodevelopmental consequences. However, the ABCD study’s longitudinal framework—tracking participants from late childhood through early adulthood—offers a unique opportunity to delineate trajectories of brain connectivity and behavior over time. As future releases include additional waves of imaging and behavioral data, longitudinal modeling can elucidate how PCE influences the evolution of functional network organization and cognitive-emotional outcomes. Building on our prior work applying connectivity-based analytic frameworks to developmental datasets, future research will be well-positioned to construct comprehensive models linking PCE to dynamic brain–behavior pathways across adolescence.

## Supplementary Material

This is a list of supplementary files associated with this preprint. Click to download.

• SI.docx

• SupplementalMaterial.docx

## Data Availability

Data used in the preparation of this article were obtained from the ABCD study via NIH Brain Development Cohorts (NBDC) Data Sharing Platform (https://www.nbdc-datahub.org/).
